# Multiplex PCR Assay for the Identification of Four Species of the *Anopheles* Leucosphyrus Sub-Group in Malaysia

**DOI:** 10.3390/insects13020195

**Published:** 2022-02-13

**Authors:** Sandthya Pramasivan, Jonathan Wee Kent Liew, Nantha Kumar Jeyaprakasam, Van Lun Low, Romano Ngui, Indra Vythilingam

**Affiliations:** 1 Department of Parasitology, Faculty of Medicine, University Malaya, Kuala Lumpur 50603, Malaysia; sandthya96@gmail.com (S.P.); jonathan_liew@nea.gov.sg (J.W.K.L.); j_nanthakumar@hotmail.com (N.K.J.); romano@um.edu.my (R.N.); 2 Environmental Health Institute, National Environment Agency, Singapore 228231, Singapore; 3 Tropical Infectious Diseases Research & Education Centre (TIDREC), University Malaya, Kuala Lumpur 50603, Malaysia; vanlun_low@um.edu.my; 4 Malaria Research Centre, Faculty of Medicine and Health Sciences, University Malaysia Sarawak (UNIMAS), Kota Samarahan 94300, Malaysia

**Keywords:** species identification, multiplex PCR assay, *Anopheles*, simian malaria, *ITS2*, Malaysia

## Abstract

**Simple Summary:**

*Plasmodium* parasites cause malaria. The bites of infected female *Anopheles* mosquitoes, known as “malaria vectors,” transmit the parasites to people. To prevent the spread of malaria, precise mosquito species identification is essential. This study aims to develop a quick and accurate method for identifying the *Anopheles* species (*An. introlatus*, *An. latens*, *An. cracens,* and *An. balabacensis*), which have been incriminated as vectors for simian malaria in Malaysia. Overall, six primers targeting the internal transcribed spacer 2 (*ITS2*) region of each species were designed for this assay. This study is helpful for the researchers or vector-related field workers to correctly identify the mosquitoes for control activities.

**Abstract:**

The Leucosphyrus Group of mosquitoes are the major simian malaria vectors in Malaysia. Accurate species identification is required to help in curbing the spread of simian malaria. The aim of the study is to provide an accurate molecular method for identifying the four important *Anopheles* vector species found in Malaysia. Mosquito specimens were collected from various localities in Malaysia, where simian malaria cases were reported. DNA from 122 mosquito specimens was tested to develop a multiplex polymerase chain reaction (PCR) assay. The specificity of this assay was tested against other mosquito species. Molecular identification of the species was further confirmed by analysing the internal transcribed spacer 2 (*ITS2*) DNA region of the specimens. *Anopheles balabacensis* and *An. latens* showed two distinct clades in the phylogenetic tree. The multiplex PCR assay was developed based on the *ITS2* region for the identification of *Anopheles introlatus* (298–299 bp), *Anopheles latens* (197–198 bp), *Anopheles cracens* (421–426 bp), and *Anopheles balabacensis* (224–228 bp). This method will be useful to accurately identify the major *Anopheles* Leucosphyrus Group species in Malaysia, which are difficult to identify morphologically, to determine the correct vector as well as its geographical distribution.

## 1. Introduction

Malaria continues to be a public health issue in many tropical countries, especially Africa [1], while in the Asian region, malaria was eradicated from Sri Lanka in 2016 [2] and recently from China [3]. Malaysia is in the pipeline for malaria elimination as no indigenous human malaria cases have been reported since 2018 [4]. However, in Southeast Asia, humans have been infected with *Plasmodium knowlesi*, a simian malaria. [5]. Moreover, *P. cynomolgi* [6,7,8,9,10] and *P. inui* have also been reported in humans [11,12]. In Malaysia, *P. knowlesi* is the predominant species affecting humans, and 3212 cases have been reported in 2019 [13]. The *Anopheles* Leucosphyrus Group of mosquitoes has been incriminated as the vectors of simian malaria [14]. There are 20 named species under the Leucosphyrus Group in the Neomyzomyia series [15,16]. This group is further classified into the Hackeri, Leucosphyrus, and Riparis subgroups. The Leucosphyrus sub-group consists of Dirus (seven sibling species) and Leucosphyrus (five sibling species) complexes [17,18] which are morphologically similar in all life phases [15,17,18]. Seven species, classified into three subgroups mentioned above, exist in Malaysia. The major simian malaria vectors of Malaysia belonging to the *Anopheles* Leucosphyrus Group are as follows: Leucosphyrus complex (*An. latens*, *An. introlatus*, *An. balabacensis* [19,20,21]); Dirus complex (*An. cracens* [22]); and Hackeri sub-group: *An. hackeri* [23]. Members of the Riparis sub-group *An. macarthuri* and *An. pujutensis* (Hackeri sub-group) are found in Malaysia but have not been incriminated as vectors thus far, although *An. pujutensis* has been suspected as a vector [14].

In the fight against malaria, one of the greatest challenges is the identification of these vectors based on morphological characteristics. Since each species has a unique role in malaria transmission, phenotypic misidentification is likely to have a significant influence on malaria vector strategy and control in specific areas [18,24,25,26,27,28,29]. Misidentification of these species can be a significant issue to the public health authorities performing vector control activities. For example, *An. introlatus* had been mistakenly recognised as *An. latens* morphologically [30]. Both species occur sympatrically and are difficult to be identified morphologically [17,31,32,33]. As a result, other techniques are required to identify these species.

Mosquitoes belonging to species complexes can be differentiated using various methods such as cross-breeding experiments, electrophoretic variation at enzymes loci, chromosome banding patterns, and molecular investigations [29]. Currently, molecular analysis to identify the species (using multiplex PCR assays) is often carried out in laboratories. This is because correct species identification is required for studies such as vector incrimination, pesticide susceptibility evaluations, and vector geographical distribution.

The tools for precise identification of the Leucosphyrus Group of *Anopheles* mosquitoes, which are simian malaria vectors, are not well established in Malaysia. This is due to the fact that these species had a very minor role in human malaria transmission in Peninsular Malaysia, albeit a major vector in Malaysian Borneo (*An. balabacensis* and *An. latens*). However, *An. dirus* complex has been well studied and plays a major role in human malaria transmission in the Greater Mekong Region [34]. The *An. dirus* complex can be distinguished by polytene, mitotic chromosomes, isoenzyme electrophoresis, DNA probes, PCR-RFLP, and non-radioactive DNA hybridisation [35,36,37,38,39,40,41,42,43]. However, these techniques have major drawbacks that limit their extensive usage in research because they require specific technical skill and knowledge for cytotaxonomy test, frozen material needed for isozymes, a large amount of DNA such as three to five mosquitoes per well for PCR-RFLP, or they allow the identification of only one, two or three species in DNA probes or non-radioactive DNA hybridisation. Hence, multiplex PCR has been developed to identify the *An. dirus* complex [44,45]. Despite the availability of these identification techniques for vector epidemiological studies, a simpler and more robust methodology is required.

Thus, the aim of this study is to create a fast and accurate identification method to distinguish four species of the major Leucosphyrus Group of *Anopheles* mosquitoes in Malaysia, i.e., *An. introlatus*, *An. latens*, *An. cracens,* and *An. balabacensis.* The use of molecular markers is more field-friendly for identifying anopheline mosquitoes because very little tissue (e.g., legs of the mosquitoes) is required.

## 2. Materials and Methods

### 2.1. Sample Collection

**Figure 1:**
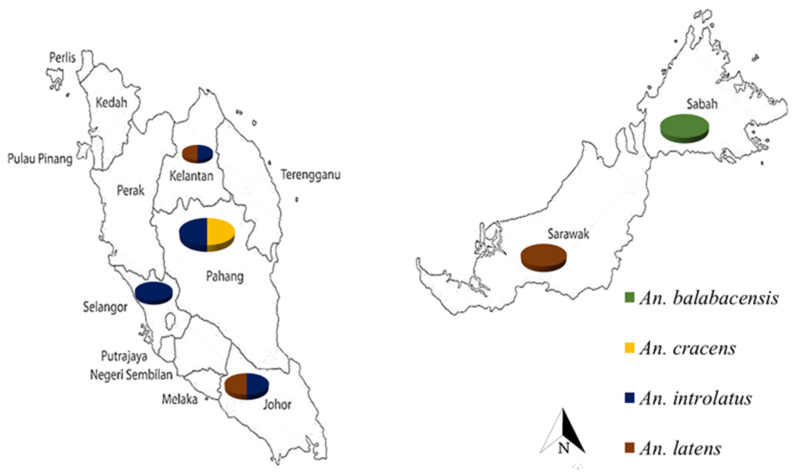
Mosquito sampling sites by species collected from Peninsular Malaysia and Malaysian Borneo (Sabah and Sarawak).

Four species of female *Anopheles* mosquitoes, namely *An. balabacensis*, *An. introlatus*, *An. cracens*, *An. latens* clade I and *An. latens* clade II [46] used in the study were collected from different states in Malaysia, namely Johor, Kelantan, Pahang, Selangor, Sabah, and Sarawak, where most simian malaria infections were reported [47] (Figure 1) (Table S1). Mosquitoes were collected using bare-leg capture (BLC) [48] as well as human baited trap, CDC light trap, and mosquito magnet from 18:00 to 23:30 as described in the work of [49]. The *Anopheles* mosquitoes were morphologically identified using the keys of Reid [50] and Sallum [18].

### 2.2. DNA Extraction and Molecular Identification of Field Caught Anopheles

DNA was extracted from the mosquitoes’ legs using either InstaGene Matrix (Bio-Rad, Hercules, CA, USA) or DNeasy^®^ tissue Kit (Qiagen, Germany) according to the manufacturers’s protocol. The extracted DNA was kept at −20 °C until required. All *Anopheles* mosquitoes from the Leucosphyrus Group obtained in this study, including some archive samples, were further molecularly characterised using the internal transcribed spacer 2 (*ITS2*) region and mitochondrial cytochrome c oxidase subunit I (*COI*) gene. The *ITS2* was amplified by ITS2A and ITS2B primers [24], the PCR conditions were as follows: denaturation at 95 °C for 2 min, 35 cycles of amplification at 95 °C for 30 s, annealing step at 51 °C for 30 s with elongation step at 72 °C for 1 min, followed by final elongation step of 10 min at 72 °C. LCO1490 and HCO2198 primers [29] were used to amplify the *COI* gene. The PCR conditions were as follows: denaturation at 95 °C for 3 min, 35 cycles of amplification at 95 °C for 1 min, annealing step at 50 °C for 1 min with elongation step at 72 °C for 1 min, followed by final elongation step of 10 min at 72 °C and held at a temperature of 4 °C. Each reaction mixture of 25 μL contained 5 μL DNA template, 0.5 μM primers, respectively, 0.2 mM dNTP, 3 mM MgCl_2_, 1 × GoTaq^®^ Flexi Buffer, and 1.0 U of GoTaq^®^ DNA polymerase (Promega Corporation, Madison, WI, USA). This master mix was used for both primer sets. Amplicons were subjected to electrophoresis on 1.5% agarose gels. The amplified product was purified from the gel and sequenced. All the sequences were checked against those in the Gene Bank using BLAST. A species is confirmed by ≥98% identity percentage and query coverage to the deposited sequence.

### 2.3. Sequence Analysis

The *ITS2* sequences from representative *An. balabacensis*, *An. cracens*, *An. introlatus* and *An. latens* samples collected from different areas were used. Sequences were aligned with other deposited sequences obtained from the NCBI Gene Bank using BioEdit (Version 7.2). MEGA–X (Version 10.1.8) was used to generate a phylogenetic tree using maximum-likelihood (ML) with 1000 bootstrap replicates. Sequences obtained from this study were deposited in the NCBI Gene Bank *An. cracens*, MZ575625-MZ57532; *An. introlatus*, MZ575650-MZ575658; *An. latens*, MZ575633-MZ575635, MW587948-MW587950.

### 2.4. Primer Design

*ITS2* DNA sequences were used to design *Anopheles* species-specific primers in this study [44,45,46,47,48,49,51,52,53,54,55,56,57]. Single-round multiplex PCR was designed based on species-specific variations in the sequences of the *ITS2*, a ribosomal DNA gene (rDNA) commonly used to distinguish cryptic *Anopheles* species, particularly those belonging to Asian complexes and groups [44,45,51,52,53,54,55,56,57]. Previously published *ITS2* sequences and *ITS2* sequences obtained from this study were included: *An. balabacensis* (KY883194-KY883201, KC508607-KC508611, JQ424794.1-JQ424825, MG008613-MG008624), *An. introlatus* (MG008577-MG008586, KM032613), *An. cracens* (KJ462197-KJ462201, MG008561-MG008576) and *An. latens* (MG008596-MG008612, MW587948-MW587956). The sequences were aligned using Clustal Omega software programme to obtain a consensus sequence for each species. Then, these species-specific consensus sequences were aligned together, and specific sites for primer design were manually selected for each species. After selection of the regions, primers were designed using PrimerOuest^TM^ tool programme. For the four *Anopheles* species found in Malaysia, a universal forward primer and species-specific reverse primers were developed.

### 2.5. Multiplex PCR Assay for Four Anopheles Species

Each primer was tested with 122 mosquito samples comprising of the four species from the Leucosphyrus group (*An. balabacensis* (21), *An. cracens* (25), *An. introlatus* (30) and *An. latens* (23), as well as with other mosquito species (two each of *An. dirus*, *An. maculatus*, *An. donaldi*, *An. minimus*, *An. barbirostris*, *An. sinensis*, *An. aconitus*, and three each *of Ae. aegypti*, *Ae. albopictus*, and *Armigeres subalbatus*) to check the length of the amplified fragment and determine primer specificity. Molecularly confirmed species were used as positive controls. Non-template control (NTC) was used as a negative control. The PCR was carried out using 25 µL volume containing 1 unit of GoTaq^®^ G2 Flexi DNA Polymerase, 1× GoTaq^®^ Flexi Buffer, 1.5 mM MgCl_2_ (Promega, Madison, WI, USA), 0.2 µM dNTP, each primer at 0.1 µM and 2 µL of extracted DNA. The PCR conditions were as follows: 95 °C for 2 min, followed by 40 cycles at 95 °C for 30 s, and 68 °C for 1 min 15 s, with a final extension step at 72 °C for 10 min and held at a temperature of 4 °C. The PCR products were subjected to electrophoresis on a 1.5% agarose gel.

## 3. Results

**Figure 2:**
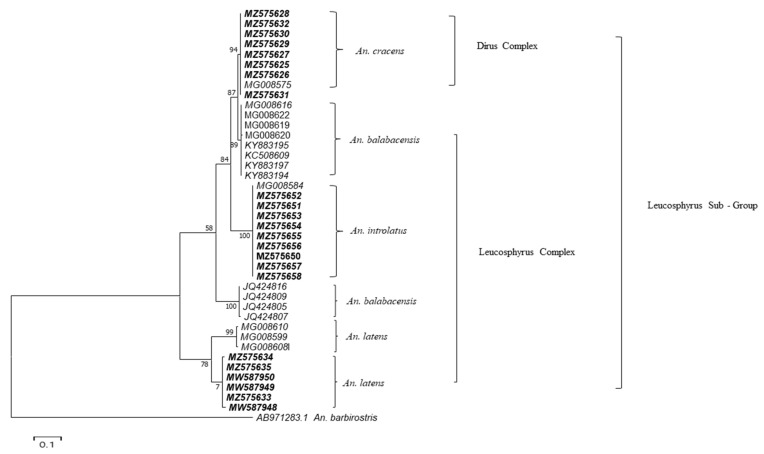
Phylogenetic tree of *Anopheles* Leucosphyrus group specimens sampled from six localities in Malaysia conScheme 2. sequence. The sequences in bold were deposited by this group, while unbold sequences are from Gene Bank.

Phylogenetic tree based on the ML approach showed that the *An. introlatus* and *An. cracens* collected in different areas where known malaria cases occurred formed their respective monophyletic clade (Figure 2). However, *An. balabacensis* and *An. latens* formed more than one clade. *An. latens* associated with the populations from East and West Malaysia were observed in the tree constructed from the *ITS2.*

Six primers were developed based on the *ITS2* sequence alignment (Table 1), and the specificity of each primer was tested.

**Figure 3:**
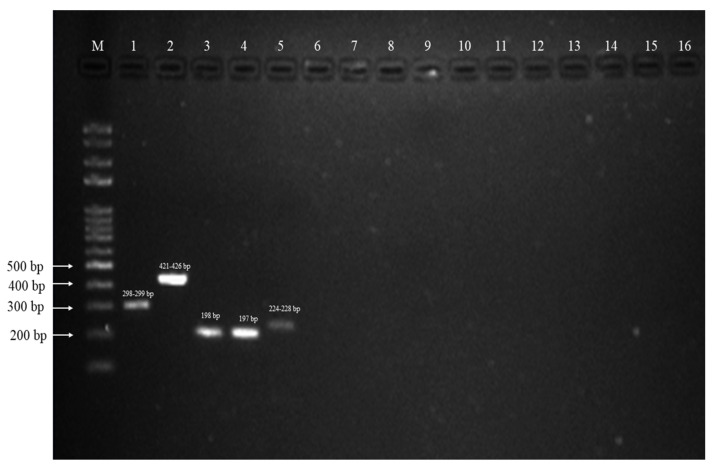
Products from the multiplex PCR run on a 1.5% agarose gel. M, 100 bp plus ladder; lane 1, *An. introlatus* (298–299); lane 2, *An. cracens* (421–426); lane 3, *An. latens* clade I (198); lane 4, *An. latens* clade II (197); lane 5, *An. balabacensis* (224–228); lane 6, *An. dirus*; lane 7, *An. minimus*; lane 8, *An. maculatus*; lane 9, *An. donaldi*; lane 10, *An. barbirostris*; lane 11, *An. sinensis*; lane 12, *An. aconitus*; lane 13, *Ar. subalbatus*; lane 14, *Ae. aegypti*; lane 15, *Ae. albopictus;* and lane 16, negative control.

The gel image (Figure 3) shows the specificity of the multiplex primers where only samples from the Leucosphyrus Group had been amplified while mosquitoes species from other groups were not successfully amplified by the designed primers.

*Anopheles* *introlatus* had 100% positive amplification. However, some of the samples tested were from DNA that was extracted some years ago, especially *An. balabacensis* and *An. latens* were not positive, perhaps due to the degradation of DNA. On the other hand, DNA extraction of old samples of whole mosquitoes preserved at −20 °C yielded positive bands by multiplex PCR (Table 2).

## 4. Discussion

The geographical range of Leucosphyrus Group includes southwestern India eastwards to southern China, Taiwan, Southeast Asia’s mainland, Indonesia, and the Philippines. [54,58]. Morphological criteria for the Leucosphyrus Group are sometimes difficult to be applied due to overlapping morphological characteristics. In order to study vectors in the light of control activities, correct identification of species and subsequent monitoring of *Anopheles* spp. is required to understand their spatial distributions, larval habitats, and population dynamics. Although the major important characteristics for species identification are scales on wings (wing patterns) and scales on legs, it is difficult to apply if the characters are destroyed during collections or storage [18,54]. For example, when a portion of the hindtarsome 4 is missing with pale scales at the base, females of *An. introlatus* can be incorrectly identified as *Anopheles nemophilous* of the Dirus complex [18]. Furthermore, the morphology of *An. balabacensis* is highly polymorphic, and thus characters used to differentiate the species between Leucosphyrus and Dirus complex, which are the presence of basal pale scales on hindtarsomere 4 and the existence of accessory sector pale (ASP) spots on vein C, subcosta, and R are important [59].

Currently, molecular markers are being used to solve identification problems. For accurate species identification, molecular markers, particularly the rRNA *ITS2* gene, are used to distinguish between sibling species in several Asian *Anopheles* complexes such as *An. minimus* group [46], *An. hycarnus* group [47], *An. dirus* [45], *An. maculatus* [48], and *An. fluviatilis* [49]. The developed multiplex PCR in this study is useful in differentiating all four major Leucosphyrus species found in Malaysia. The multiplex PCR was validated on 122 specimens collected throughout Malaysia with a wide sampling range (Figure 1). The forward primer in our technique is universal, but the reverse primers are species specific. Molecular tools are more convenient in field studies since specimens could be dried and usually need only a small amount of tissue, such as mosquito legs.

Furthermore, the two *An. latens* clades that cannot be differentiated by the multiplex PCR assay based on PCR product size, are most likely linked to two different geographical regions, i.e., Peninsular Malaysia and Malaysian Borneo. Genetic variation was observed between *An. latens* from Peninsular and Borneo Malaysia. This may be due to the geographical separation between Peninsular Malaysia and Malaysian Borneo. Landscape factors influence the natural environment of species, resulting in genetic differences among the same species [60]. *ITS2* is a well-acknowledged molecular marker for mosquito taxonomy as well as would be more reliable as a phylogenetic marker among closely related species [61,62]. However, to determine whether this species constitutes a cryptic species, a detailed morphological analysis is required for the *An. latens* from the two clades. Genetic diversity can be seen in the species complex. *Anopheles balabacensis*, Baisas is a complex species that has been recorded in a few countries. The two clades of *An. balabacensis* could be due to the cryptic diversity of that species [63]. However, the *An. balabacensis* primer was designed on the conserved region of sequences of both clades, which are predominant in Malaysia, as well as reliable sequences deposited in Gene Bank. A reverse primer for *An. nemophilous* species from the Malaysian Leucosphyrus Group has been developed and is awaiting validation pending the availability of field samples.

Accurate *Anopheles* species identification is essential in malaria vector surveillance because it influences the control intervention as well as pesticide product selection. Precise species identification allows for evaluations of vector competence, insecticide susceptibility, and important behavioural characteristics such as feeding and resting behaviours by species, leading to the development of insecticide-based control strategies that can be supplemented by additional malaria elimination techniques [57].

Proper species identification, involving both morphological and molecular techniques, is vital for species confirmation and vector ecology and for instituting effective control measures [64]. A few studies have demonstrated the importance of correct species identification in vector surveillance programmes. *Anopheles minimus*, a main malaria vector in India, was morphologically mistaken as *An. fluviatilis*, but each species was accurately identified using PCR of the *ITS2* region [65]. If species are identified wrongly, it would be a serious problem, especially if it is a vector. Misidentification of *Anopheles* species could result in the wrong application of vector control measures [29,66]. *Anopheles vaneedeni* was also identified as a new malaria vector in South Africa during a malaria monitoring programme that used the *ITS2* region for specific identification [67]. The technique suggested here allows for quick and reliable identification by requiring only PCR and electrophoresis of individual specimens. Identification of species for distribution purposes would aid in understanding the malaria distribution pattern as well as the development of vector and malaria preventative measures.

To reduce misidentification of the Malaysian Leucosphyrus Group of mosquitoes, a few steps must be taken. The first step is to correctly identify the samples morphologically. To avoid any misunderstanding regarding vector status, an *ITS2* and cytochrome c oxidase subunit I (*COI*) sequence confirmation of the mosquito sample is required. Given the wide geographical distribution of the Leucosphyrus Group of mosquitoes and the involvement of plasmodia transmission, it is important to better know the exact diversity of species that comprise the complex as well as which ones are vectors of public health importance so that control efforts can be better targeted to maximise transmission suppression and increase the chances of malaria elimination [68]. As a result, an accurate identification approach based on molecular techniques is required to distinguish between species, allowing for further research into their bionomics, distribution, and role in disease transmission. The species of the mosquitoes cannot be determined solely through gel electrophoresis and estimation of the sizes of the PCR products unless the primers are species specific.

This current assay will be useful for molecular identification for the scenario where sequencing was not performed for the study. To our knowledge, this is the first multiplex PCR designed to identify the four simian malaria vectors of the Leucosphyrus Group of *Anopheles* mosquitoes in Malaysia. Previous studies have used the universal *ITS2* A and *ITS2* B primers to identify the species based on band size without sequencing [69]. This approach may not be accurate because the band size can be the same across species, especially when using universal primers [70]. It is essential to perform molecular identification to determine the species of the mosquitoes because misidentification can be detrimental. Many malaria control laboratories are already equipped to perform PCR tests; therefore, no new equipment will be required to identify, in this case, the Leucosphyrus Group species. The multiplex PCR test is fast, inexpensive, specific, and easy to use. Only a small amount of material (1–2 legs) is required for identification, leaving the remainder of the body parts available for further analysis, such as sporozoite detection, blood meal analysis, population genetics, or pesticide resistance status.

With *P. knowlesi* being the predominant species and with *P. cynomolgi* and *P. inui* affecting humans in Malaysia [5], it is vital to study the distribution and to correctly identify the vectors responsible for transmission. Accurate identification of vectors and their distribution for simian malaria parasites is essential to eventually help to implement malaria elimination strategy accordingly. Thus, this multiplex PCR technique will aid in the development of current knowledge on the species distribution of Leucosphyrus Group mosquitoes in large surveys of anopheline populations and large collections in Malaysia. Furthermore, since this technique does not rely on skilled interpretation, there is no subjective bias in the identification.

## 5. Conclusions

This study provides a quick, fast, specific, and effective multiplex PCR test for detecting the four Leucosphyrus Group species (*An. balabacensis*, *An. cracens*, *An. introlatus,* and *An. latens*) known to be simian malaria vectors. This is a trustworthy identification technique that will enable a wide variety of investigations on the Leucosphyrus Group of species. The multiplex PCR can aid in identification if sequencing technology is not available. Correct species identification is critical in all downstream works concerning the species in question, especially for malaria vector control programmes. Further research work can be performed on more *Anopheles* species whose identities are reliably confirmed or developing complete primer sets for *Anopheles* Leucosphyrus Group of mosquitoes in Malaysia and the region.

## Figures and Tables

**Table 1:** Universal forward primer and the five *Anopheles* species-specific reverse primers (from this study) for *An. latens*, *An. introlatus*, *An. cracens*, and *An. balabacensis* with the sequences and the product size.

	Primer’s Name	Sequences	Product Sizes (bp)
Universal forward primer	LeucogrpFwd	5′-GCG YCG CTG GCC TGC ACG-3′	-
*An. balabacensis*	balabaRev	5′-CGG CGC AGC GAC TCY ACC G-3′	224–228
*An. cracens*	craRev4	5′-GC ACC GCT CTT GGC GGG ATA T-3′	421–426
*An. introlatus*	introRev3	5′-CG ACG AGC GCG YGA GCG A-3′	298–299
*An. latens* Clade I	laten1Rev	5′-CCC GGG CGT CCG GTG TTT-3′	198
*An. latens* Clade II	laten2Rev	5′-CCG GGC GTC YGC GGT GTA C-3′	197

**Table 2:** Results from multiplex PCR on sequenced samples of Leucosphyrus Group of mosquitoes.

Species	No. of Specimen	No. of Sequenced Samples	Positive in Multiplex PCR Assay	% Positive Amplification
*An. balabacensis*	21	21	18	85.71
*An. cracens*	25	25	23	92
*An. introlatus*	30	30	30	100
*An. latens*	23	23	21	91.30

## Data Availability

All data are available within the manuscript.

## Supplementary Materials

The following supporting information can be downloaded at: https://www.mdpi.com/2075-4450/13/2/195/s1, Table S1: Details of *Anopeheles* Leucosphyrus Group of mosquitoes used in this study.
