# Correction: Pramasivan et al. Multiplex PCR Assay for the Identification of Four Species of the *Anopheles* Leucosphyrus Sub-Group in Malaysia. *Insects* 2022, *13*, 195

**DOI:** 10.3390/insects13050466

**Published:** 2022-05-17

**Authors:** Sandthya Pramasivan, Jonathan Wee Kent Liew, Nantha Kumar Jeyaprakasam, Van Lun Low, Romano Ngui, Indra Vythilingam

**Affiliations:** 1 Department of Parasitology, Faculty of Medicine, University Malaya, Kuala Lumpur 50603, Malaysia; sandthya96@gmail.com (S.P.); jonathan_liew@nea.gov.sg (J.W.K.L.); j_nanthakumar@hotmail.com (N.K.J.); romano@um.edu.my (R.N.); 2 Environmental Health Institute, National Environment Agency, Singapore 228231, Singapore; 3 Tropical Infectious Diseases Research & Education Centre (TIDREC), University Malaya, Kuala Lumpur 50603, Malaysia; vanlun_low@um.edu.my; 4 Malaria Research Centre, Faculty of Medicine and Health Sciences, University Malaysia Sarawak (UNIMAS), Kota Samarahan 94300, Malaysia

## Error in Table

In the original publication [1], there is a mistake in Table 1 as published. The sequence for *An. balabacensis* balaba Rev was written as 5′-GC ACC GCT CTT GGC GGG ATA T-3′ 224–228 bp. The correct Table 1 appears below. The authors apologize for any inconvenience caused and state that the scientific conclusions are unaffected. This correction was approved by the Academic Editor. The original publication has also been updated.

## Figures and Tables

**Table 1:** Universal forward primer and the five *Anopheles* species-specific reverse primers (from this study) for *An. latens*, *An. introlatus*, *An. cracens*, and *An. balabacensis* with the sequences and the product size.

	Primer’s Name	Sequences	Product Sizes (bp)
Universal forward primer	LeucogrpFwd	5′-GCG YCG CTG GCC TGC ACG-3′	-
*An. balabacensis*	balabaRev	5′-CGG CGC AGC GAC TCY ACC G-3′	224–228
*An. cracens*	craRev4	5′-GC ACC GCT CTT GGC GGG ATA T-3′	421–426
*An. introlatus*	introRev3	5′-CG ACG AGC GCG YGA GCG A-3′	298–299
*An. latens Clade* I	laten1Rev	5′-CCC GGG CGT CCG GTG TTT-3′	198
*An. latens* Clade II	laten2Rev	5′-CCG GGC GTC YGC GGT GTA C-3′	197
