# Supplementary material for: Multiplex PCR Assay for the Identification of Four Species of the Anopheles Leucosphyrus Sub-Group in Malaysia
Source: Insects. 2022 Feb 13;13(2):195. doi: 10.3390/insects13020195 (PMC8878329; doi:10.3390/insects13020195)
Supplement: Supplementary file 1 [file insects-13-00195-s001.zip › insects-1532753-supplementary.pdf]

Table S1: Details of *Anopeheles* Leucophyrus group of mosquitoes used in this study

Species	States	Numbers	Year of collection	Collection methods sequence	Accession number
An. balabacensis	Sabah	21	2013-2014	HLC	-
An. cracens	Pahang	25	2011;2019	HLC & mosquito magnet	MZ575625 – MZ575632
An. introlatus	Selangor	5	2012	HLC	-
	Pahang	5	2020	HLC & mosquito magnet	
	Johor	20	2019-2020	HLC, CDC light trap, HBT & mosquito magnet	MW587822 -MW587832; MZ575650 – MZ575658
An. latens	Sarawak	7	2020	HLC	-
	Kelantan	8	2019	HLC	MW587948 - MW587956;
	Johor	8	2019	HLC	MZ575633 – MZ575635
